# Exploring factors influencing skin incision to the delivery time and their impact on neonatal outcomes among emergency cesarean deliveries indicated for non-reassured fetal heart rate status

**DOI:** 10.3389/fped.2023.1224508

**Published:** 2023-09-21

**Authors:** Dereje Zewdu, Temesgen Tantu, Fikretsion Degemu, Michael Hawlet, Nitsuh Dejene, Emebet Asefa

**Affiliations:** ^1^ Department of Anesthesia, College of Medicine and Health Science, Wolkite University, Wolkite, Ethiopia; ^2^ Department of Obstetrics and Gynecology, College of Medicine and Health Science, Wolkite University, Wolkite, Ethiopia; ^3^ Department of Pediatrics and Child Health, College of Medicine and Health Science, Wolkite University, Wolkite, Ethiopia

**Keywords:** fetal distress, emergency cesarean delivery, incision-to-delivery time, adverse neonatal outcomes, NICU

## Abstract

**Background:**

The time interval between skin incision and delivery (S-D) is crucial in determining neonatal outcome; however, little is known about the influencing factors and their impact on neonatal outcomes, particularly among emergency cesarean deliveries (ECD) indicated for fetal distress. This study investigated the factors influencing S-D time and their effects on neonatal outcomes among mothers who underwent ECD for non-reassured fetal heart rate status.

**Methods:**

This retrospective cohort study involved 426 mother-infant pairs over four years. We retrieved data from the medical records, including baseline characteristics, perioperative data, and neonatal outcomes. Using multivariable logistic regression analysis, adjusted odd ratios, and a 95% confidence interval, potential factors influencing S-D time and their impacts on neonatal outcomes were assessed. A *p*-value of less than 0.05 was considered statistically significant.

**Results:**

Factors independently associated with longer S-D time (>8 min) were mothers who had previous CD (AOR 5.9: 95% CI 2.2–16.1), obese mothers (AOR 6.2: 95% CI 1.6–24.5), and the second stage of labor (AOR 5.3: 95% CI 2.4–11.7). Adverse neonatal outcomes, including a 5th minute Apgar score of less than 7, the need for NICU admission, and neonatal death, were significantly higher in the longer S-D time interval [47.7% vs. 8.9%; *p*-value 0.001], [21.9% vs. 9.1%; *p*-value 0.001], and [32% vs. 11.8%; *p*-value = 0.004], respectively. Obese mothers and the second stage of labor, but not previous CD, adversely impact neonatal outcomes.

**Conclusion:**

Longer S-D times are significantly associated with adverse neonatal outcomes. Factors that prolong the time interval between skin incision and delivery may or may not necessarily be associated with adverse neonatal outcomes. Considering surgical techniques that shorten the incision-delivery time and preparation for advanced neonatal care for risky subjects would help reduce detrimental neonatal consequences.

## Introduction

Cesarean delivery (CD) has increased globally and accounts between 25% to 33% of all births in Ethiopia, and about 19% of their indications are associated with fetal distress or non-reassuring fetal heart rate patterns (1).

Fetal distress is a critical incident requiring immediate CD (2). Study demonstrated that shortening the decision-to-delivery time in such conditions is believed to improve early neonatal outcomes and reduce the number of neonates admitted to the neonatal intensive care unit (3). However, there is inconclusive data concerning the impact of a longer skin incision-to-delivery (S-D) time interval on neonatal outcomes. While some studies (4–6) have found a significant association between an increased S-D time interval and adverse neonatal parameters, others (7–10) demonstrate no association between these variables. Surprisingly, there is no published data on the impact of prolonged S-D time on the neonatal outcomes of emergency CD, particularly for non-reassured fetal heart rate (NRFHR) indications.

Previous studies investigating determinant factors for longer S-D time, previous CD, increasing body mass index (BMI), fetal weight, the stage of labor, surgical adhesions, and the level of obstetricians experience are reported as potential factors (7–9, 11–13); however, the negative impacts of these factors on the neonatal outcomes remain unexplored. These indicates a delay in S-D time may be associated with untoward neonatal consequences, particularly the potentially prolonged time without fetal monitoring following induction of anesthesia or incision-to-delivery of the neonates because of unanticipated critical events such as uterine hypoperfusion.

Nevertheless, factors that increase the S-D time may or may not be directly associated with adverse neonatal outcomes (14–16). Thus, exploring and understanding the factors that prolonged the incision and delivery time and their impact on neonatal outcomes would provide more insight into improving clinical practice.

In low-resource settings, where the burden of emergency cesarean delivery and adverse maternal and neonatal outcomes remains a significant problem (17, 18), there is insufficient data on the association between skin incision-to-delivery time interval and neonatal outcomes, and there are no guidelines adopted into clinical practice.

To better understand, further investigations are needed to determine the influencing factors and the association between longer S-D time interval and adverse neonatal outcomes. These would help develop an intervention approach and translate the findings into clinical practice to minimize the negative consequences for neonatal outcomes.

Our aim, therefore, was to identify the risk factors that prolong the time interval between skin incision and delivery and their impact on the immediate neonatal outcomes in women who underwent emergency cesarean delivery for the indication of a non-reassured fetal heart rate in Wolkite University Specialized and Teaching Hospital in Southern Ethiopia.

## Methods and materials

### Study design, study population and study period

This retrospective study included all data for mother-infant pairs who underwent emergency cesarean delivery (ECD) indicated for a non-reassured fetal heart rate pattern at Wolkite University Specialized and Teaching Hospital, Ethiopia, over four years, from September 2019 to February 2023. The study included 432 lower uterine segment CDs indicated for NRFHR patterns among 2340 emergency CDs for singleton term gestations carried out during the study period. NRFHR patterns constitute fetal heartbeat greater than 160 and less than 120 beats per minute lasting for 10 min using cardiotocography. We excluded participants with multiple pregnancies, still births, incomplete documentation, and unknown or other than 37–42 weeks of gestation.

The Wolkite University Ethical Review Board provided approval for the study. Due to the retrospective nature of the study design, we were exempted by the Ethical review board of Wolkite University from receiving informed written consent for data collection. This study was conducted per the Declaration of Helsinki Ethical Principles for Medical Research Involving Human Subjects Protocol.

### Data collection

We retrospectively collected perioperative data from the medical records and obstetric operation notes on maternal age, referral status, parity, type of anesthesia (general vs. spinal), BMI, history of previous CD, maternal medical illness, and time interval (minutes) between skin incisions and delivery of the neonates. Maternal medical illness is mothers who had previous history of diabetes mellitus, chronic hypertension, asthma and others before the pregnancy. We extracted the neonatal data from the neonate medical records, including gestational age, birth weight, neonatal sex, the 5th minute Apgar score, the need for NICU admissions, and neonatal death.

### Statistical analysis

Data were coded and transported into SPSS (Statistical Package for the Social Sciences for Windows version 26.0, SPSS Inc., Chicago, Illinois, USA) for analysis.

Results were converted into categorical variables and displayed as frequency tables. The association between independent and outcome variables was analyzed using the Chi-square and Fisher exact tests. All influencing factors for the S-D time interval were analyzed using bivariate analysis, and the variables with a *p*-value < 0.25, were entered into a multivariable logistic regression analysis. Multivariable logistic regression analysis to check the association between potential factors influencing the time interval between skin-incision and delivery, the impact of longer S-D time on neonatal outcomes, and the association between factors prolonging S-D time with adverse neonatal outcomes, including the 5th minute Apgar score < 7, the need for NICU admissions, and neonatal death within 7 days after delivery. The results of independent factors were presented as a frequency table, odds ratio (OR), and adjusted odds ratio (AOR) with a 95% confidence interval (CI). A *p*-value of <0.05 was considered statistically significant.

### Perioperative and neonatal intensive unit care practice

Our hospital's resident or senior obstetricians on duty typically use a fetoscope and Cardiotocography (CTG) in selected cases to diagnose non-reassured fetal heart rate or fetal distress. Fetal distress (Non reassuring fetal status) is defined as fetal heartbeat patterns above 160 beats per minute (BPM) and less than 120 BPM lasting for 10 min. The conservative interventions for fetal distress are fluid resuscitation, face mask oxygen supplementation for the mother, changing maternal position, antipyretics if there is any record of maternal fever, and discontinuing any drug that can result in fetal heartbeat abnormalities. If there is no response to the above measures, then the diagnosis of fetal distress would be entertained. As soon as fetal distress became evident, the obstetric staff members informed the mothers of the circumstances and secured their written consent for undergoing emergency CD. The obstetric team comprises an obstetrician, anesthetist, midwife, and the operating room nursing staff.

On the arrival of the mothers to the operating theater, the anesthesia team applied standard monitoring and provided anesthesia care per the institution's protocol. Based on the mother-infant's physiologic status and the clinical judgment of the experienced anesthesia specialists and obstetrician's on-duty determined the types of anesthesia, whether spinal or general. Intraoperatively, the time interval between skin incision and delivery of the neonate, 1st and 5th minute Apgar scores, neonatal sex, and neonatal weight were registered on standardized anesthesia notes. Based on the clinical judgment of the senior anesthetist, obstetricians, and other resuscitation teams, the neonates needed NICU admission.

In our NICU setting, the expertise involved and the number of staff, medical supplies, and overall infrastructure are substandard. The unit includes pediatricians, resident pediatricians, medical interns, nurses, and other supporting staff. Nurses and resident pediatricians are available 24 h a day; the attending senior pediatrician supervises the overall clinical activities in the unit. However, there are no neonatologists or well-trained neonatal nurses; the nurses serving in this unit received training limited to regular neonatal care.

Our NICU room has a separate room for a triage area, a critical room with six beds each for term and preterm neonates, an isolation room with six beds, and a waiting area for families of newly admitted neonates. The unit provides critical care for neonates diagnosed with hypothermia, hypoglycemia, respiratory distress, perinatal asphyxia, feeding difficulties, and neonates requiring phototherapy and positive pressure ventilation.

The decision to discharge the admitted neonates depends upon the improvement or resolution of the underlying indications for admission, the ability to breastfeed independently, regulate temperature at room temperature, regain their target weight, and maintain oxygen saturation without ventilation support.

## Results

During the study period, of the 472 participants who underwent emergency CD for NRFHR patterns, 426 (%) were included in the final analysis. Results for baseline characteristics are displayed in Table 1.

**Table 1:** Baseline characteristics of the study participants.

Characteristics		Range
Maternal age, years	26.3 ± 4.8	18–43
Maternal age > 35 years	29 (6.8)	
Previous CD	103 (24.3)	1–3
BMI, kg/m^2^	27.9 ± 2.6	21–37
BMI > 30 kg/m^2^	79 (18.6)	
First delivery	165 (38.9)	
Maternal medical illness	12 (2.8)	
Stage of labor at delivery		
First stage	206 (48.6)	
Second stage	218 (51.4)	
Type of anesthesia		
Spinal anesthesia	392 (92.5)	
General anesthesia	32 (7.5)	
Skin incision to delivery, minutes	5.6 ± 1.7	3–15
Skin incision to delivery > 8 min	55 (13)	
Referred from other centers	163 (38.4)	
Operated on duty hours	244 (57.5)	
Neonate		
Non-cephalic presentations	52 (12.3)	
Birth weight, gram	3,307 ± 347	2,300–4500
Birth weight > 4,000 grams	29 (6.8)	
Gestational age, weeks	38.6 ± 1.3	37–42
Sex (Male)	234 (55.2)	
5th minutes Apgar score < 7	44 (10.4)	
Need of NICU admission	128 (30.2)	
Neonatal death	25 (5.9)	

NICU, neonatal intensive care unit; CD, cesarean delivery; BMI, body mass index; Kg/m^2^, kilogram per meter square.

The average maternal age and body mass index were 26.3 ± 4.8 and 27.9 ± 2.6, respectively. About one-fourth of mothers had previous CD history, and 38.9% were having their first delivery. Maternal medical illness was rare, with only 2.8% having such a history. An average time interval between skin incision and delivery of the neonates was 5.6 ± 1.7 min, and about 55 (13%) had S-D times of more than 8 min. Most study participants underwent ECD at the second stage of labor (51.4%), received spinal anesthesia (92.5%), and 38.4% were referred from other centers. With regards to adverse neonatal outcomes, the 5th minute Apgar score < 7, the need for NICU admission, and neonatal death were observed in (10.4%), (30.2%) and (5.9%), respectively.

Bivariate analysis as shown in (Table 2), S-D interval > 8 min was significantly associated with previous CD (25.3% vs. 9.1%; *p*-value < 0.001), BMI (34.2% vs. 8.5% and 5.9%; *p*-value < 0.001), stage of labor (19.7% vs. 5.8%; *p*-value < 0.001), mothers referred from other centers (17.2% vs. 10.3%; *p*-value = 0.042), non-cephalic presentation (23.1% vs. 11.6%; *p*-value = 0.021) and fetal weight > 4,000 grams (20.7% vs. 12.4%; *p*-value = 0.2) compared to S-D ≤ 8 min group.

**Table 2:** Bivariate analysis to identify the relationship between influencing factors with skin incision-to-delivery time interval of ≤8 min and >8 min.

Characteristics	S-D ≤ 8 min	S-D > 8 min	*p*-value
Maternal age > 35 years			0.48
Yes	24 (82.7)	5 (17.3)	
No	345 (87.4)	50 (12.6)	
Previous CD			<0.001*
Yes	77 (74.7)	26 (25.3)	
No	292 (90.9)	29 (9.1)	
BMI, kg/m^2^			<0.001*
<24.9	48 (94.1)	3 (5.9)	
25–29.9	269 (91.5)	25 (8.5)	
>30	52 (65.8)	27 (34.2)	
First delivery			0.47
Yes	146 (88.5)	19 (11.5)	
No	233 (86.6)	36 (13.4)	
Maternal medical illness			0.21
Yes	9 (75)	3 (25)	
No	360 (87.4)	52 (12.6)	
Stage of labor at delivery			<0.001*
First stage	194 (94.2)	12 (5.8)	
Second stage	175 (80.3)	43 (19.7)	
Type of anesthesia			0.31
Spinal anesthesia	343 (87.5)	49 (12.5)	
General anesthesia	26 (81.25)	6 (18.75)	
Referred from other centers			**0** **.** **042**
Yes	135 (82.8)	28 (17.2)	
No	234 (89.7)	27 (10.3)	
Operated time			0.28
On working hours	153 (85)	27 (15)	
On duty hours	216 (88.5)	28 (11.5)	
Non-cephalic presentations			**0** **.** **021**
Yes	40 (76.9)	12 (23.1)	
No	329 (88.4)	43 (11.6)	
Birth weight > 4,000 grams			0.2
Yes	23 (79.3)	6 (20.7)	
No	346 (87.6)	49 (12.4)	
Sex of the neonate			0.1
Male	198(84.6)	36(15.4)	
Female	171(90)	19(10)	

* Strong association.

The bold values are represents is association.

S-D time, skin incision to delivery time; CD, cesarean delivery; BMI, body mass index; Kg/m2, kilogram per meter square.

Factors significantly associated with a longer S-D time undergo further analysis to identify independent factors influencing the incision-to-delivery time interval.

Multivariable logistic regression analysis showed that obese mothers (BMI > 30 kg/m^2^) were 5.38 times (AOR = 5.38, 95% CI 1.4–20.6) more likely to have longer S-D times than mothers with BMIs of 25–29.9 and less than 24.9. The risk of a longer S-D time was 4.85 times (AOR = 4.85, 95% CI 2.37–9.92) higher in mothers with previous histories of CD than in mothers without CD. Mothers who underwent emergency CD during the second stage of labor were 5.6 times (AOR = 5.6, 95% CI 2.5–12.36) more likely to have a longer S-D time than mothers who underwent emergency CD during the first stage of labor. Furthermore, the likelihood of a longer S-D time was more than 2-fold (AOR = 2.2, 95% CI 0.7–6.7) higher in mothers who gave birth with a fetal weight > 4,000 grams, which is insignificant as shown in Table 3.

**Table 3:** Multivariable logistic regression analysis identifying potential factors influencing skin-incision-to-delivery time during emergency cesarean delivery.

Characteristics, (%)	S-D ≤ 8 min	S-D > 8 min	COR (95% CI)	AOR (95% CI)	*p*-value
Previous CD					
Yes	77 (74.7)	26 (25.3)	3.4 (1.9–6.2)	4.85 (2.37–9.92)	<0.001*
No	292 (90.9)	29 (9.1)	1	1	
BMI, kg/m^2^					
<24.9	48 (94.1)	3 (5.9)	1	1	
25–29.9	269 (91.5)	25 (8.5)	1.5 (0.4–5.1)	1.1 (0.26–3.71)	0.98
>30	52 (65.8)	27 (34.2)	8.3 (2.4–29.1)	5.38 (1.4–20.6)	**0** **.** **014**
Maternal medical illness					
Yes	9 (75)	3 (25)	2.3 (0.6–8.8)	1.56 (0.27–9.13)	0.62
No	360 (87.4)	52 (12.6)	1	1	
Stage of labor at delivery					
First stage	194 (94.2)	12 (5.8)	1	1	
Second stage	175 (80.3)	43 (19.7)	4.1 (2.1–7.7)	5.6 (2.5–12.36)	<0.001*
Referred					
Yes	135 (82.8)	28 (17.2)	1.8 (1.1–3.2)	1.7 (0.89–3.25)	0.12
No	234 (89.7)	27 (10.3)	1	1	
Non-cephalic presentations					
Yes	40 (76.9)	12 (23.1)	2.3 (1.2–4.7)	2.2 (0.9–5.1)	0.059
No	329 (88.4)	43 (11.6)	1	1	
Birth weight > 4,000 grams					
Yes	23 (79.3)	6 (20.7)	1.8 (0.7–4.8)	2.2 (0.7–6.7)	0.15
No	346 (87.6)	49 (12.4)	1	1	
Sex of the neonate					
Male	198 (84.6)	36 (15.4)	1.3 (0.7–2.2)	1.7 (0.9–3.5)	0.19
Female	171(90)	19(10)	1	1	

* Strong association.

The bold values are represents is association.

COR, crude odd ratio; AOR, adjusted odd ratio; CI, confidence interval; CD, cesarean delivery; BMI, body mass index; Kg/m^2^, kilogram per meter square.

The prevalence of a 5th-minute Apgar score of less than 7, neonates needing NICU admission, and neonatal death were 10.33%, 30.04%, and 5.87%, respectively. The proportion of 5th minute Apgar scores less than 7, neonates needing NICU admission, and neonatal death were significantly high in the S-D time > 8 min group, [47.7% vs. 8.9%; *p*-value < 0.001], [21.9% vs. 9.1%; *p*-value < 0.001], and [32% vs. 11.8%; *p*-value = 0.004], respectively (Table 4).

**Table 4:** The impact of longer skin-incision to delivery time interval (>8 min) on neonatal outcomes.

Adverse neonatal outcomes, (%)	S-D ≤ 8 min	S-D > 8 min	*p*-value
5th minutes Apgar score < 7			<0.001*
Yes	23 (52.3)	21 (47.7)	
No	346 (91.1)	34 (8.9)	
Need of NICU admission			<0.001*
Yes	100 (78.1)	28 (21.9)	
No	269 (90.9)	27 (9.1)	
Neonatal death within 7-days			**0** **.** **004**
Yes	17 (68)	8 (32)	
No	352 (88.2)	47(11.8)	

* Strong association.

The bold values are represents is association.

S-D, skin incision to delivery; NICU, neonatal intensive care unit.

We also evaluated the relationship between adverse neonatal outcomes and the factors associated with a longer S-D time interval, as shown in Table 5. Logistic regression analysis found that mothers with previous CD had an insignificant association with all adverse neonatal outcomes (*p*-value > 0.05). However, while the second stage of labor significantly increases the number of neonates requiring NICU admissions (*p*-value = 0.006), obesity is significantly associated with adverse neonatal outcomes, including a 5th minute Apgar score less than 7 (*p*-value = 0.002), the need for NICU admission (*p*-value = 0.001), and neonatal death (*p*-value = 0.035).

**Table 5:** Logistic regression analysis to identify the relationship between adverse neonatal outcomes and factors associated with longer S-D time.

Neonatal outcomes	Odd ratio 95 CI	*p*-value
5th minutes Apgar score < 7		
Obesity (BMI > 30 kg/m^2^)	2.6 (1.5–4.7)	**0** **.** **002**
Second stage of labor	1.6 (0.9–2.7)	0.1
Previous CD	1.4 (0.7–2.5)	0.28
NICU admission		
Obesity (BMI > 30 kg/m^2^)	2,6 (1,6–4,4)	**<0** **.** **001**
Second stage of labor	1.8 (1.2–2.8)	**0** **.** **006**
Previous CD	1.1 (0.7–1.8)	0.64
Neonatal death		
Obesity (BMI > 30 kg/m^2^)	3.1 (0.6–15.2)	**0** **.** **035**
Second stage of labor	2.1 (0,9–4.9)	0.09
Previous CD	1.5 (0.6–3.6)	0.36

The bold values are represents is association.

CD, cesarean delivery; BMI, body mass index.

## Discussion

The current study demonstrated that factors associated with a longer skin-incision-to-delivery time were obese mothers (BMI > 30 kg/m^2^), previous CD history, and the second stage of labor. We also observed a significant association between longer S-D times > 8 min and adverse neonatal outcomes, including a 5th-minute Apgar score < 7, the need for NICU admission, and neonatal death within 7 days among women who underwent emergency CD for the indication of non-reassured fetal heart rate status.

Controversy exists in previous studies concerning the association between the S-D interval and adverse neonatal outcomes. Some studies (7–9) demonstrated no association between the S-D interval and neonatal outcomes. These could be attributed to the studies focused on women who underwent elective CDs, where unfavorable neonatal outcomes were rare, and the heterogeneity of the study participants. While the present study included women undergoing emergency CD for non-reassured fetal status indications only, this could explain the observed differences.

Unsurprisingly, numerous studies showed a significant association between longer S-D intervals and more detrimental newborn outcomes, consistent with our findings (4–19). However, some factors influencing S-D time have no impact on neonatal outcomes. For instance, having a previous CD does not affect neonatal outcomes, despite being significantly associated with longer S-D intervals, as revealed in the current study. The study observed that mothers with previous CD histories were 4.85 times more likely to be associated with a longer S-D time than mothers without CD histories. Findings from other studies (7–9, 12, 13, 20, 21) also demonstrated that the likelihood of longer S-D time intervals was significantly higher in mothers with previous CDs because the proportion of adhesions increased as the number of CDs increased. Moreover, a recent study (16) conducted in 2023 showed that time from incision to neonatal delivery has no effect on neonatal outcomes in women with one or more previous cesarean sections, supporting our findings. Mothers with prior CD tend to receive close observation and timely intervention, leading to fewer neonatal complications.

Some of the variables, including stage of labor and obesity, were found to be linked with adverse neonatal outcomes. CDs performed during the second stage of labor were a critical factor associated with detrimental neonatal consequences (22–24). CS performed at the second stage of labor is technically challenging to shorten the delivery time and associated with higher odds of neonatal complications, including a 5th minute Apgar score of less than 7, an increased need for NICU admission, and neonatal death. This is likely due to the deeply engaged and impacted fetal head in the pelvis results the difficulty to remove the fetus from the uterus and took longer times than CS performed at first stage of labor (24, 25). Thus, anticipating such challenges would help take into account alternative approaches to mitigate the risk of increased incision-to-delivery time.

In the second stage of labor, the deeply impacted fetal head in the pelvis, extensive uterine contractions, and typically thinned uterus increase neonatal complications, including a 5th minute Apgar score of less than 7, an increased need for NICU admission, and neonatal death. Thus, anticipating such challenges would help to consider alternative approaches that mitigate the risk of increased delivery time. Similarly, obese mothers with a BMI greater than 30 kg/m^2^ were 5.38 times more likely to be associated with longer S-D time intervals. In agreement with our findings, previous studies (5–7, 9) also observed a linear relationship between BMI and the S-D time interval. Conner et al. (14) demonstrated that increasing BMI is not only associated with a significantly prolonged skin incision to delivery time but also with neonatal morbidity, in agreement with our findings. Despite close observation and advanced neonatal intensive care throughout perioperative period is imperative for obese mothers second stage CD, whether these factors negatively affect neonatal outcome because of the prolong the S-D time or independently need further investigations.

There are some limitations in the present retrospective study of prospectively collected data. Although the study included all emergency cesarean deliveries indicated for NRFHR, it failed to determine the role of other underlying pathologies of fetal distress on neonatal outcomes. Critical factors, including the interval between uterine incisions and neonatal delivery and the presence of post-CD adhesions, were excluded from the study because they were not documented in their medical records. Also, neonatal acid-base status is not studied because the laboratory setup for investigating neonatal cord PH analysis was inaccessible in the study setting.

Future prospective studies, which include the time interval between induction of anesthesia and uterine incision to delivery, adhesions, and other underlying obstetric pathologies for NRFHR, and cord PH analysis, would be helpful for measuring their respective impacts on neonatal outcomes.

## Conclusion

Previous CD, obesity, and the second stage of labor were significantly associated with a longer S-D time interval. However, obesity and the second stage of labor are significantly associated with adverse neonatal outcomes, not previous CD. Therefore, although longer S-D times were significantly associated with fatal neonatal outcomes, identifying peculiar factors and their impact on neonatal outcomes would be helpful. Considering various strategies to reduce the time interval between skin incision and delivery and providing timely and intensive care in anticipated cases is imperative to minimize the consequences of adverse neonatal outcomes.

## Data Availability

The raw data supporting the conclusions of this article will be made available by the authors, without undue reservation.
